# Humerus Brace for Treating Humeral Shaft Fractures: A Retrospective Cohort Study of Union and Failure Rates

**DOI:** 10.7759/cureus.96897

**Published:** 2025-11-15

**Authors:** Mahmoud Mersal, Safa Yousif, Omer Zubair, Shady Salama, Mohamed Elgendy, Osama Embaby, Abdelrahman Embabi, Serajdin Ajnin

**Affiliations:** 1 Trauma and Orthopaedics, University Hospitals Birmingham (UHB) NHS Foundation Trust, Birmingham, GBR; 2 Trauma and Orthopaedics, Royal Free London, NHS Foundation Trust, London, GBR; 3 Trauma and Orthopaedics, Farooq Hospital, Lahore, Lahore, PAK; 4 Trauma and Orthopaedics, Sandwell and West Birmingham Hospitals NHS Trust, Birmingham, GBR; 5 Emergency Medicine, Royal Surrey NHS Foundation Trust, London, GBR

**Keywords:** conservative management, functional brace, humeral shaft fracture, non-union, union rate

## Abstract

Background

Non-operative management of humeral shaft fractures, which is a common challenging upper limb injury, using a functional brace, remains widely practiced. However, modern reported outcomes vary, and actual union rates may differ from the historic benchmarks.

Objective

To evaluate the union rate and overall success of humeral shaft fractures treated conservatively in a functional brace, and to analyse how fracture level and pattern may influence outcomes.

Methods

This retrospective audit included adult patients with closed humeral shaft fractures managed in a functional brace between January 2012 and January 2019. The study was conducted at University Hospitals Birmingham NHS Foundation Trust, including two major teaching hospitals, and was registered under the institutional clinical governance system (Audit Code: CARMS-19262). Data were obtained from orthotics records across both sites. Demographics, fracture characteristics, complications, and outcomes were analysed. Failure was defined as surgical fixation for malalignment or non-union, or persistence of non-union at final follow-up.

Results

Eighty-eight patients met the inclusion criteria (mean age 61.8 years, 58% female). Union was achieved in 78.4% of cases, and the overall success rate, defined as union without subsequent surgery, was 67 %. Conversely, 33% of patients (n=29) failed brace treatment, with 25% (n=22) ultimately requiring surgical fixation. Proximal and mid-shaft fractures showed higher rates of delayed or non-union compared with distal shaft fractures. Fracture pattern (transverse, oblique, spiral, comminuted) did not significantly influence union or failure.

Conclusion

Functional bracing achieved a modest union rate compared with historical data. Fracture level, particularly proximal and mid-shaft involvement, was associated with a significantly higher rate of non-union. Future studies should assess the influence of patient compliance, initial alignment, and brace fit on union potential.

## Introduction

Since the original description by Sarmiento et al., functional bracing has remained the cornerstone of non-operative treatment for humeral shaft fractures [1]. The technique was designed to allow early mobilisation while maintaining alignment through circumferential compression of the soft tissues. Most contemporary studies have reported good-to-excellent outcomes in over 80 % of patients, with union rates approaching 94-95 %; however, clinical results have become increasingly variable, with reported union rates ranging from 65% to 95% in modern series [2-6].

More recent literature suggests that outcomes under real-world conditions may be less favourable, with reports of non-union rates higher than anticipated [7]. In parallel, advances in surgical fixation, including plating and intramedullary nailing, have offered alternatives with potentially faster healing and lower failure rates, but at the cost of surgical risks [8,9].

Variation in non-operative outcomes may relate to differences in patient selection, fracture morphology, compliance, and local practice. Despite widespread acceptance, there remains debate about which fracture types benefit most from conservative treatment and when early surgical fixation should be considered [10,11].

Within this evolving landscape, it remains clinically important to characterise how effective functional bracing truly is in modern practice, particularly in the UK NHS context, and to identify which fracture characteristics correlate with poorer outcomes. To this end, this audit aimed to evaluate the union rate and functional success (union without surgery) of humeral shaft fractures treated with a brace, and to examine how fracture level (proximal, mid, or distal) and fracture pattern influence these outcomes.

## Materials and methods

This audit was conducted at the University Hospitals Birmingham NHS Foundation Trust, including two major teaching hospitals, over the period January 2012 to January 2019, under registration number CARMS-19262.

Adult patients (≥16 years) with closed humeral shaft fractures, defined as fractures of the humeral diaphysis excluding the proximal metaphysis and distal humerus, who were managed conservatively with a standard functional (Sarmiento-type) humeral brace after an initial splint/coaptation phase, were identified via orthotics and radiology records.

Exclusion criteria comprised open fractures, pathological fractures, patients initially planned for surgical fixation, early death (<4 weeks), or insufficient radiographic/follow-up data to assess union.

We collected demographic data (age, sex, side of injury), fracture level (proximal third of shaft, mid-shaft, distal third of shaft), fracture pattern (transverse, oblique, spiral, comminuted), complications (radial nerve palsy, skin irritation), time to radiographic union, and need for conversion to surgery.

Union was defined as radiographic bridging callus on at least three cortices with the absence of pain at the fracture site and restoration of limb function. Failure of brace treatment was defined as the need for surgical fixation for malalignment/non-union, or persistent non-union at final follow-up without surgical conversion.

Data were analysed to calculate the union rate and the success rate. Associations between fracture level/pattern and outcome were examined using chi-square tests (p < 0.05 considered significant).

## Results

A total of 117 patients were initially identified across the two hospital sites. After applying the inclusion and exclusion criteria, 88 patients were eligible for analysis. The remaining 29 patients were excluded for the following reasons: two were treated in plaster slab or cast, five underwent open reduction and internal fixation (ORIF) within one week of injury, two were lost to follow-up, four died within four weeks of the fracture, six were under 16 years of age, and ten did not provide consent for data analysis.

The mean age of the included cohort was 61.8 ± 20.7 years (range 16-98), with a slight female predominance (58 % female, n = 51; 42 % male, n = 37). The left arm was affected in 68.2 % of patients (n = 60), while 31.8 % (n = 28) sustained right-sided fractures.

Proximal-third shaft fractures were the most common, accounting for 47.7 % (n = 42), followed by mid-shaft fractures (42.0 %, n = 37) and distal-third fractures (10.2 %, n = 9). Fracture configuration was variable: Twenty-five percent transverse, 25 % oblique, 31.8 % comminuted, and 18.2 % spiral. Baseline characteristics are summarised in Tables 1-2.

**Table 1:** Demographic characteristics

Variable	Value
Age (years), mean ± SD (range)	61.8 ± 20.7 (16–98)
Sex: Male n (%)	37 (42%)
Sex: Female n (%)	51 (58%)
Side: Right n (%)	28 (31.8%)
Side: Left n (%)	60 (68.2%)

**Table 2:** Fracture characteristics

Characteristic	Value
Fracture level – Proximal n (%)	42 (47.7%)
Fracture level – Mid-shaft n (%)	37 (42.0%)
Fracture level – Distal n (%)	9 (10.2%)
Fracture pattern – Transverse n (%)	22 (25.0%)
Fracture pattern – Oblique n (%)	22 (25.0%)
Fracture pattern – Spiral n (%)	16 (18.2%)
Fracture pattern – Comminuted n (%)	28 (31.8%)

Union and delayed/non-union

Radiographic union was achieved in 69 of 88 patients (78.4 %). Nineteen patients (21.6 %) developed delayed or non-union. Of these, 12 required surgical fixation, while 7 remained ununited at the final follow-up but did not undergo surgery due to medical contraindications or personal refusal.

The analysis showed a statistically significant association between fracture level and delayed/non-union (p = 0.038). When examined individually, proximal (p = 0.011) and mid-shaft (p = 0.036) fractures demonstrated significantly higher non-union rates compared with distal fractures. In contrast, fracture pattern was not significantly associated with non-union (p > 0.05) (Table 3).

**Table 3:** Fracture level vs union outcome Chi-square p = 0.038 (overall); proximal p = 0.011, mid-shaft p = 0.036.

Level	Union (n)	Non-union / Delayed union (n)	Total (n)
Distal	8	1	9
Mid-shaft	33	4	37
Proximal	28	14	42
Total	69	19	88

Brace failure and surgical conversion

Brace treatment was deemed successful in 59 of 88 patients (67 %). The remaining 29 patients (33 %) met the criteria for brace failure, defined as surgical fixation for non-union or malalignment, or persistent non-union at final review.

A total of 22 patients (25 %) underwent surgical fixation-10 for unacceptable alignment and 12 for non-union or delayed union. Among these, two required multiple procedures for persistent non-union. Of those who had surgery, 36.4 % (8/22) were treated with intramedullary nails, and 63.6 % (14/22) underwent plate fixation. Seven patients (8 %) remained ununited but were managed non-operatively due to comorbidity or patient preference.

No statistically significant relationship was observed between fracture pattern and brace failure (p > 0.05). Similarly, age, sex, and fracture level were not significantly associated with treatment failure (Tables 4-5).

**Table 4:** Treatment outcomes

Outcome	Frequency (n)	Percent (%)
No failure (healed in brace)	59	67.0
Surgery for unacceptable alignment	10	11.4
Surgery for non-union / delayed union	12	13.6
Non-union, no surgery	7	8.0
Total	88	100.0

**Table 5:** Fracture pattern vs brace outcome p > 0.05 for the association between pattern and brace failure.

Fracture pattern	Union (n)	Surgery for malalignment (n)	Surgery for non-union (n)	Non-union, no surgery (n)	Total (n)
Comminuted	19	2	3	4	28
Oblique	11	6	2	3	22
Spiral	12	0	4	0	16
Transverse	17	2	3	0	22

Complications

Of the 66 patients who completed conservative management in a brace, 7 remained non-united at last follow-up. Two patients presented with radial nerve palsy prior to brace application, both of which persisted to some degree. One of these underwent neurolysis during subsequent surgical fixation. Additionally, one patient developed a skin allergy attributed to the brace material, which resolved after adjustment and topical treatment. No new radial nerve injuries were identified following brace application.

Summary

Functional bracing achieved a union rate of 78.4% and a functional success rate of 67 %. Proximal and mid-shaft fractures were significantly more likely to progress to delayed or non-union, whereas fracture pattern, age, and sex did not significantly affect the outcome. Complications were rare, limited mainly to pre-existing nerve injuries and minor skin irritation.

## Discussion

This retrospective audit demonstrated a union rate of 78.4 % and a functional success rate (union without surgery) of 67 % for humeral shaft fractures managed conservatively with a functional brace. While these figures reaffirm the ongoing role of non-operative management, they are lower than the classic results reported by Sarmiento et al., who achieved a union rate exceeding 90 % with functional bracing [1]. More recent studies have shown a wide variation in outcomes, with reported union rates ranging from 65 % to 95 %, depending on patient selection, fracture configuration, and compliance [2-6].

The difference between historical and modern results likely reflects changing patient demographics and real-world practice. Early series typically included younger, fitter patients with simple, mid-diaphyseal fractures and high compliance with follow-up [1,2]. In contrast, contemporary practice, particularly within the NHS, often involves older, more comorbid, or less mobile patients, and many proximal or comminuted fractures are now trialled in a brace before proceeding to surgery [7,11]. These factors collectively explain why modern functional bracing studies report lower union rates than the original Sarmiento cohort.

Randomised and prospective studies have increasingly compared bracing with surgical fixation. The FISH randomised clinical trial demonstrated that around one-third of patients treated with a brace ultimately required surgical fixation, though functional outcomes at one year were similar between groups [8]. Matsunaga et al. conducted another randomized controlled trial (RCT) comparing functional bracing to minimally invasive bridge plating and found earlier recovery and lower non-union rates with surgery [12]. Similarly, recent papers confirmed that operative fixation, either plating or intramedullary nailing, achieves faster recovery and significantly reduces non-union risk, though at the cost of surgical complications such as infection and nerve injury [9,13].

Our results align with these findings. Fracture level was significantly associated with delayed or non-union, with proximal and mid-shaft fractures showing poorer healing compared with distal fractures. These findings echo the biomechanical rationale that the proximal and mid-diaphyseal regions experience higher torsional stresses and less cortical contact area, which compromises fracture stability within the brace [14,15]. In contrast, fracture pattern (transverse, oblique, spiral, or comminuted) did not significantly influence healing in our series, consistent with previous evidence that geometry alone is a weak predictor of non-union once alignment and soft-tissue compression are adequate [3-6].

Functional bracing remains a valuable first-line strategy, especially for distal-third fractures and minimally displaced mid-shaft injuries, but should be approached with caution in proximal fractures and in patients with known risk factors for poor healing, including smoking, diabetes, and obesity [16,17]. Operative management generally provides earlier functional recovery and lower rates of non-union, but the long-term differences in function often diminish after one year, suggesting that patient-specific factors should drive decision-making [8,12,13,18].

Clinical implications

From a clinical perspective, these results emphasise the importance of setting realistic expectations when recommending functional bracing. Patients with proximal or mid-shaft humeral fractures should be counselled that the risk of delayed or non-union, and the potential need for secondary surgery, is considerably higher than in distal fractures. While functional bracing remains a low-risk and cost-effective treatment option, particularly for lower-demand or high-surgical-risk patients, it requires close radiological and clinical follow-up.

Clinicians should monitor for early indicators of delayed healing, such as persistent pain, functional limitation, or absent radiographic callus formation at 12-16 weeks, and should not hesitate to consider timely conversion to surgical fixation when indicated.

This audit also highlights the value of incorporating standardised radiographic indices (e.g., fracture gap, displacement ratio) and compliance monitoring to guide patient selection and predict outcomes more reliably.

Limitations

This study is limited by its retrospective nature and lack of standardised radiographic displacement or compliance data. Furthermore, granular data on patient-specific co-morbidities known to affect bone healing, such as smoking status, diabetes, or BMI, were not consistently available in the audited records and could not be analysed. The absence of functional outcome scores such as DASH or Oxford Shoulder Score also restricts comparison with prospective series. However, it provides valuable real-world data from a large NHS cohort and complements the findings of recent RCTs and meta-analyses by highlighting which fracture types perform less favourably in a brace.

Future prospective studies should incorporate standardised radiographic measures of displacement and objective assessments of brace compliance to better define predictors of success.

## Conclusions

Functional bracing achieved union in about three-quarters of humeral shaft fractures, with two-thirds healing successfully without surgery. Proximal and mid-shaft fractures showed a higher risk of delayed or non-union, whereas the fracture pattern had no significant effect. These findings highlight the importance of careful patient selection, close follow-up, and early surgical consideration in higher-risk fractures.
